# Does the fortified milk with high iron dose improve the neurodevelopment of healthy infants? Randomized controlled trial

**DOI:** 10.1186/s12887-019-1679-0

**Published:** 2019-09-05

**Authors:** Lucía Iglesias Vázquez, Josefa Canals, Núria Voltas, Cristina Jardí, Carmen Hernández, Cristina Bedmar, Joaquín Escribano, Núria Aranda, Rosa Jiménez, Josep Maria Barroso, Blanca Ribot, Victoria Arija

**Affiliations:** 1 Unit of Preventive Medicine and Public Health, Faculty of Medicine and Health Science, Universitat Rovira i Virgili, Reus, Spain; 2 CRAMC (Centre de Recerca en Avaluació I Mesura de la Conducta), Unit of Psychology, Universitat Rovira i Virgili, Tarragona, Spain; 3 Unit of Pediatrics, Faculty of Medicine and Health Science, Hospital Universitari Sant Joan de Reus, Universitat Rovira i Virgili, Reus, Spain; 4 IISPV (Institut d’Investigació Sanitària Pere Virgili), Reus, Spain; 5 IDIAP (Institut Universitari d’Investigació en Atenció Primària) Jordi Gol, Barcelona, Spain

**Keywords:** Anthropometry, Child health, Development, Iron supplementation, Lactation

## Abstract

**Background:**

Since iron plays an important role in several physiological processes, its deficiency but also overload may harm the development of children. The aim was to assess the effect of iron–fortified milk on the iron biochemical status and the neurodevelopment of children at 12 months of age.

**Methods:**

Randomized controlled trial conducted in 133 Spanish children, allocated in two groups to receive formula milk fortified with 1.2 or 0.4 mg/100 mL of iron between 6 and 12 months of age. Psychomotor (PDI) and Mental (MDI) Development Index were assessed by the Bayley Scales before and after the intervention. Maternal obstetrical and psychosocial variables were recorded. The biochemical iron status of children was measured and data about breastfeeding, anthropometry and infections during the first year of life were registered.

**Results:**

Children fortified with 1.2 mg/100 mL of iron, compared with 0.4 mg/100 mL, showed higher serum ferritin (21.5 vs 19.1 μg/L) and lower percentage of both iron deficiency (1.1 to 5.9% vs 3.8 to 16.7%, respectively, from 6 to 12 months) and iron deficiency anemia (4.3 to 1.1% vs 0 to 4.2%, respectively, from 6 to 12 months) at the end of the intervention. No significant differences were found on neurodevelopment from 6 to 12 months between children who received high dose of Fe compared with those who received low dose.

**Conclusion:**

Despite differences on the iron status were observed, there were no effects on neurodevelopment of well–nourished children in a developed country after iron supplementation with doses within dietary recommendations. Follow–up studies are needed to test for long–term neurodevelopmental improvement.

**Trial registration:**

Retrospectively registered in ClinicalTrials.gov with the ID: NCT02690675.

## Background

Iron deficiency (ID) and iron deficiency anemia (IDA) are public health problems even in developed countries, especially during childhood [1]. The prevalence of ID ranges from 2 to 29% in Europe [2] and between 9.6 and 23.3% in Spain, depending on the age group and geographical area [3, 4].

Babies are born with high iron stores [5, 6], which decline progressively during the first 6 months of life, as a result of the rapid growth of the baby [7]. Processes such as the increasing circulating blood volume, hemoglobin (Hb) formation and brain development require a great supply of iron, and turn the sixth month into a critical point in the infant’s health status [5, 8]. Some researchers argue that it is a natural process known as “physiological anemia of infancy” [7, 9]. However, because of the important role that iron plays in several physiological processes, some recent researches focus on establishing whether ID and IDA entail a pathological situation in the physical or psychological development of children [5, 10, 11]. In this regard, a recent systematic review recommends some strategies to reduce IDA in critical periods of early childhood [12].

Although several authors showed that iron fortification improves the infant’s hematological profile [13–16], a systematic review [17] warned that the evidences are inconsistent. The findings about the effect of iron fortification in young children on their neurodevelopment are also controversial. There were some evidences of a benefits on the children’s neurodevelopment and growth following the iron fortification in Chile [18], China [19], Indonesia [20] and several low–income countries, as it was shown by some systematic reviews [21]. On the contrary, some others [22–24] did not observe advantages in neurodevelopment or growth after iron intervention both in anemic and iron–sufficient healthy children. Even, Lozoff et al. [25] found worse neuropsychological scores in 10–year–old children who had been fed with formula milk fortified with high iron content (mean, 12.7 mg/L) from 6 to 12 months, compared with those who were fed with formula milk fortified with low doses of iron (mean, 2.3 mg/L). Thus, systematic reviews by Martins et al. [26] and Wang et al. [27] did not find sufficient evidence to give a definitive conclusion about the advantages and disadvantages of iron fortification in children. On the other hand, iron is an essential nutrient for the growth of some bacteria so it has been argued that ID may be a defense mechanism against some pathogens and, conversely, it is associated with a worse immune state, which may increase the susceptibility to infection [28] and, consequently, affect the child’s development.

Beyond that, the safety of the higher doses of iron (10–14 mg/L) on babies’ health is unclear as state by the ESPGHAN Nutrition Committee [29]. Given the disagreement about whether the decrease in iron levels in infants is a physiological event or a harmful occurrence, the lack of studies in developed countries and in iron replete infants, and the lack of studies with high doses of iron, our clinical trial assesses the effect of formula milk fortified with the lowest and the highest dose of iron (within the dietary recommendations) between 6 and 12 months of age on the iron–related biochemical status and on the infant’s mental and psychomotor development at 12 months.

The aim was to test the hypothesis that doses in the higher range would benefit development in infants.

## Methods

This randomized controlled trial (RCT) on iron fortification between 6 and 12 months of age was carried out in the Hospital Universitari Sant Joan de Reus (Tarragona, Spain). The study was approved by the hospital’s Ethical Committee and all parents signed an informed consent in accordance with the declaration of Helsinki. The trial was registered in ClinicalTrials.gov with the ID: NCT02690675.

### Study process

During the postpartum stay in the hospital, the parents of the children who met the inclusion criteria were informed by the researchers about the possibility of participating in the study. Inclusion criteria: gestational age ≥ 37 weeks, birthweight ≥2500 g, Caucasians and with no known disease. Exclusion criteria: iron metabolism illness, birth defects, immunodeficiency or hypothyroidism, diseases requiring intensive care, families that do not understand Catalan or Spanish and/or with very different eating habits, and having missed some of the study visits.

The intervention with iron–fortified milk was done from 6 to 12 months of age. At 1, 3 and 9 months adherence visits were scheduled. At the 6–month visit, professionals who were not members of the research group used computer programs to randomly assign the children to the low– (0.4 mg iron/100 mL) or high–iron (1.2 mg iron/100 mL) group, without taking into account any specific parameter. The randomization had a ratio of 1 (low–iron) to 3 (high–iron), based on the hypothesis that low doses of iron could be harmful to children’s health and, on the contrary, that high doses (within dietary recommendations) have been reported in previous studies certain benefits on neurodevelopment [19, 20, 30]. The type of formula milk that the babies took during the clinical trial was monitored. Formula milks were fortified by Laboratorios Ordesa S.L., and the iron content was distinguished by package color (green or red), to which clinical staff and participants were blinded. The doses were the lower and upper limit recommended by ESPGHAN [29]. All mothers were given the same food and lifestyle advice, regardless of the intervention group.

### Data collection

At birth, sociodemographic data (age, socioeconomic status [SES], parents’ education, personal and family medical history) and general characteristics of the mother and newborn (data on pregnancy, type of delivery and sex of the newborn, anthropometric measurements) were collected. The SES of the family (low, medium or high) was assessed using the Hollingshead index [31]. The mothers answered the State–Trait Anxiety Inventory (STAI) [32] to inform of the anxiety level in the pregnancy.

Forty eight hours after birth, a blood sample was taken from the infant heel to determine serum ferritin (SF). At 6 and 12 months, as well as standard clinical history data, the following measurements were taken: anthropometric details (weight, length, head circumference), cognitive development (mental and psychomotor development), and biochemistry (serum iron, serum transferrin, SF, Hb and Mean Corpuscular Volume (MCV). Aliquots of plasma and serum were stored in the hospital’s laboratory (Laboratori Biobanc–IRCIS) for subsequent measurements. The percentage of transferrin saturation (%TS) was calculated using the serum iron and serum transferrin measurements as reported in Fairbanks et al. [33] (serum iron μmol/L/serum transferrin g/L × 3.9).

In each visit, pediatricians asked the families about breastfeeding time and the number of infections that the infant may had before the visit. At 6 and 12 months, the parents answered the Parental Stress Index [34] which reports the effect that parenting has on an their stress level; for this study only the attached subscale was considered.

Since there are no clear criteria about what the normal biochemical parameters of iron status are in children, we defined the following parameters: iron stores at birth were regarded as low when SF < 25 μg/L, and at 6 and 12 months when SF < 12 μg/L, and children were considered to have ID when two or more of the following conditions were met: %TS < 16, MCV < 70 fL or SF < 12 μg/L. Children were considered to have IDA when they had ID and Hb < 11 g/dL. Further, SF is a specific marker that determines whether iron stores are depleted [35–37].

### Assessment of neurodevelopment

The Bayley Scales for Infant Development–Second Edition (BSID–II) [38] were used to assess mental and psychomotor development. The BSID provides a mental development index (MDI) (to assess memory, habituation, problem solving, early number concepts, generalization, classification, vocalizations, and language and social skills) and a psychomotor development index (PDI) (to assess the control of the gross and fine muscle groups). The BSID was administered at 6 and 12 months at the hospital by two trained developmental psychologists who had an inter–rater reliability of 90%. All the children were accompanied by at least one of the parents during the assessments. The reference population of Bayley has a mean of 100 and SD of 15, so that scores lower than 85 are defined as delayed development.

### Statistical analysis

Data are presented as percentages, means or geometric means, and standard deviations. The X^2^ test, the Student T test and the Mann–Whitney U test were used for independent samples. Non–normally distributed variables were logarithmically transformed to normalize the distributions.

Multiple linear regressions were done to explore the effect of the intervention on infant cognitive development (MDI and PDI), adjusted for gender, differences between 6 and 12 months (∆12–6 M) in head circumference, Body Mass Index and SF, mean of PSI between 6 and 12 months, MDI and PDI at 6 months and SES level.

The analyses were done with SPSS for Windows 21.0 and the significance was *p* < 0.05.

## Results

**Fig. 1:**
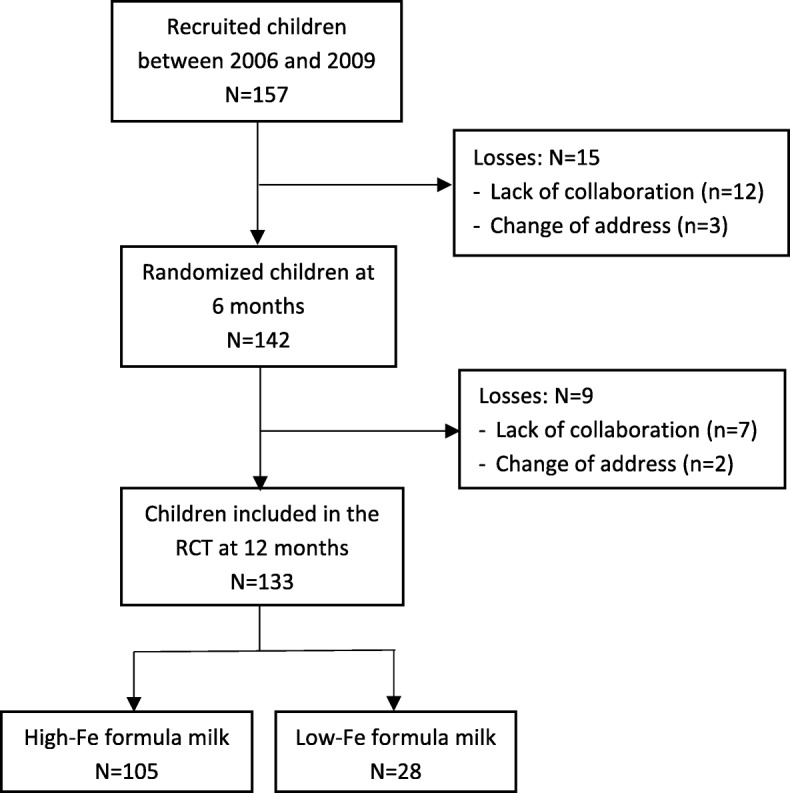
Flowchart of participants

Of a total of 157 recruited children at birth, 142 were randomized into two groups of intervention and, after the drop–out rate of 7.4% from birth to 6 months and 6.5% from 6 to 12 months, 133 were finally assessed: 105 children in the high–iron group (high–Fe) and 28 in the low–iron group (low–Fe) (Fig. 1). The loss during follow–up was mainly a lack of collaboration or absence of data. The baseline characteristics of the mothers and the infants were shown for each intervention group in Table 1. Children were non–iron–deficient at birth and had good iron stores, normal anthropometric values and also good Apgar scores. Both, mothers and children were not different according to intervention group in any of the variables, except for the high–Fe group, were mothers had higher anxiety levels and the children had greater length. The time of breastfeeding and the psychological state of the parents at 6 months was not different between iron groups. A total of 34 and 42.3% of children were breastfed or received mixed lactation at 6 months in the high–Fe and low–Fe group, respectively. The children lost (*n* = 9) have the same characteristics as participants.

**Table 1:** Baseline characteristics of the mothers and infants

	INTERVENTION		
	Formula milk (high dose of Fe) *n* = 105	Formula milk (low dose of Fe) *n* = 28	p
Mother			
Mother age, years	31.5 (4.5)	32.9 (3.9)	0.1334
Socioeconomic level, %			
Low	10.5	3.6	0.403
Medium	53.3	50	
High	36.2	46.4	
Smokers, %	21	10.7	0.179
Mode of delivery, %			
Eutocic	65.1	54.5	0.222
Forceps	16.3	9	
Caesarean	18.6	36.4	
Primiparous, %	52.1	64.3	0.268
Gestational age, weeks	39.6 (1.1)	39.4 (1.3)	0.538
STAI_State	14.4 (8.8)	11.4 (6.1)	0.056
STAI_Trait	18.1 (9.1)	13.9 (6.1)	0.028
Newborn			
Weight, g	3384.5 (408.0)	3237.7 (376.3)	0.088
Length, cm	49.9 (2.1)	48.7 (2.0)	0.009
Head circumference, cm	34.5 (1.5)	34.4 (1.7)	0.782
Body Mass Index, kg/m^2^	13.6 (1.1)	13.8 (1.3)	0.573
Serum ferritin, μg/L^#^	244.7 (1.8)	200.3 (0.5)	0.375
Presence of HFE alteration, %	33.3	28.6	0.764
Apgar 1–10	9.4 (0.4)	9.5 (0.2)	0.645
Children at 6 months			
Breastfeeding or mixed lactation, %	34.0	42.3	0.537
Time of breastfeeding, months	2.5 (2.8)	2.9 (3.0)	0.554
Total Parental Stress Index	110.42 (24.3)	111.10 (19.0)	0.892

Data were expressed in Mean (SD) or in %

*STAI* State–Trait Anxiety Inventory

^#^Geometric mean

Table 2 compares the anthropometric, biochemical and neurodevelopmental values of children from the two intervention groups at 6 and 12 months of age. The intervention with high dose of iron did not modify the anthropometrical development of children nor the infection risk. Also, the high iron formula did not improve iron levels. However, although the prevalence of SF < 12 μg/L and ID increased from 6 to 12 months in both intervention groups, this increase was lower in high–Fe group than in low–Fe group (SF < 12 μg/L: 0.4 vs 12.5, *p* < 0.001; ID: 4.8 vs 12.9, *p* = 0.053). Regarding the prevalence of IDA, it was even reduced in high–Fe group from 6 to 12 months, compared with low–Fe group (− 3.2 vs 4.2, p < 0.001). At 12 months, comparing with low–Fe group, children fed with high–Fe milk had better scores in MDI (99.1 vs 95.8) and PDI (90.8 vs 86.6), but in no case the difference was statistically significant.

**Table 2:** Characteristics of anthropometry, biochemistry and neurobehavioral development at 6 and 12 months, according to the intervention

	6 MONTHS			12 MONTHS			VARIATION FROM 6 TO 12 MONTHS ∆ 12–6 months		
	Formula milk (high dose of Fe) *n* = 105	Formula milk (low dose of Fe) *n =* 28	p	Formula milk (high dose of Fe) *n* = 105	Formula milk (low dose of Fe) *n* = 28	p	Formula milk (high dose of Fe) *n* = 105	Formula milk (low dose of Fe) *n* = 28	p
Weight, g	8051.8 (939.5)	7545.0 (715.8)	0.009	10,143.0 (1218.8)	9628.0 (1113.1)	0.048	2089.5 (562.1)	2014.0 (767.2)	0.578
Length, cm	67.7 (2.5)	66.3 (2.5)	0.011	76.2 (2.9)	74.4 (2.8)	0.007	8.5 (2.3)	8.3 (1.6)	0.636
Head Circumference, cm	43.7 (1.4)	42.9 (0.9)	0.002	46.5 (1.5)	45.7 (1.3)	0.012	2.8 (0.8)	2.8 (0.6)	0.912
Body Mass Index, kg/m^2^	17.5 (1.4)	17.2 (1.5)	0.227	17.5 (1.6)	17.4 (1.8)	0.872	−0.1 (1.1)	0.0 (1.3)	0.637
Hemoglobin, g/dL	11.7 (1.0)	11.5 (1.0)	0.479	12.0 (0.7)	11.8 (1.0)	0.218	0.4 (0.8)	0.3 (0.9)	0.557
Mean Corpuscular Volume, fl	77.5 (4.0)	78.0 (4.6)	0.589	79.0 (4.0)	78.1 (5.4)	0.344	1.4 (3.2)	0.1 (2.3)	0.073
Serum Iron, μmol/L	10.9 (4.5)	11.1 (5.2)	0.876	11.5 (4.6)	11.6 (5.0)	0.923	0.6 (5.6)	0.5 (6.4)	0.929
Serum Ferritin, μg/L^#^	27.7 (2.0)	33.4 (2.1)	0.021	21.5 (1.7)	19.1 (1.8)	0.001	−6.2 (2.0)	−14.3 (2.1)	0.055
Transferrin Saturation, %TS	16.3 (7.3)	18.7 (9.8)	0.182	17.0 (6.8)	17.1 (9.4)	0.965	0.6 (8.4)	−2.4 (7.0)	0.027
Serum Ferritin < 12 μg/L, %*	6.5	0	0.408	6.9	12.5	0.595	0.4	12.5	< 0.001
Iron Deficiency, %*	1.1	3.8	0.916	5.9	16.7	0.165	4.8	12.9	0.053
Iron Deficiency Anemia, %*	4.3	0	0.642	1.1	4.2	0.884	−3.2	4.2	< 0.001
Infections, %	3.1	2.0	0.650	25.3	25.0	0.812	22.2	23.0	0.782
Mental Development Index	94.8 (9.6)	93.0 (12.9)	0.421	99.1 (12.3)	95.8 (8)	0.217	4.6 (13.1)	2.5 (15.1)	0.509
Psychomotor Development Index	85.7 (13.9)	81.5 (10.1)	0.149	90.8 (12.8)	86.6 (11.7)	0.146	5.4 (15.7)	5.1 (12.8)	0.936

Data are expressed in Mean (SD) or in %

*In the variation from 6 to 12 months, a percentage close to zero or negative is a good result

^#^Geometric mean

Multiple regression models were performed for assessing the effect of the iron fortification on the mental (R^2^c.100 = 5.2; F92,1 = 6.025; *p* = 0.016) and psychomotor (R^2^c.100 = 11.4; F92,1 = 12.846; *p* = 0.001) development of children. The regression models were adjusted for possible confounders as follow: gender, socioeconomic level, serum ferritin and difference in BMI and head circumference from 6 to 12 months. No statistical significant effect of the intervention with formula milk fortified with high–Fe dose, compared with low–Fe dose, was found for both MDI (β = 4.53, SE = 2.89, *p* = 0.121) and PDI (β = 3.65, SE = 3.08, *p* = 0.239) in the multivariate analyses.

## Discussion

Our comprehensive evaluation of the nutritional status of children took into account both anthropometry and biochemistry from birth. We assessed iron status with a wide battery of measures. It should be borne in mind that there are no clear normality criteria on the biochemical parameters of iron status in children. The international organizations recommend having observed the combination of two or three altered parameters to determine ID and IDA. However, serum ferritin is a very specific marker, the levels of which only diminish if iron stores are depleted [35, 36, 39].

It is well documented [40–42] that SF physiologically declines throughout the first year of life. In our population, although this decrease is evident in both intervention groups from 6 to 12 months of age, it was less abrupt in children supplemented with 1.2 mg/100 mL than in those who received 0.4 mg/100 mL of iron. Similarly, even though several authors in European countries observed higher levels of SF after the intervention with iron [13]–[15, 23, 43], our results suggest that supplementation with iron doses within the dietary recommendations in healthy, well–fed children from a developed country improved their iron status, but it was not enough to replete the iron stores of children at 1 year of age. We also proposed the hypothesis, as some authors did previously [5, 10], that the physical development of children was conditioned by iron, given its implication in several physiological processes. In this case, contrary to what was hypothesized, our intervention had no effect on children’s anthropometry, which reinforce some other findings [15, 23, 24]. Recent reviews [17, 44] highlight that the knowledge about the effect of iron in the growth of children is still scarce and unclear. Beyond, the risk of infections following the iron fortification is a concern, keeping in mind that iron is an essential nutrient for the metabolism of some bacteria. About that, previous findings were in conflict [45] but our results showed that the rate of infection was not significantly higher in the children fed with high–Fe milk than in those with low–Fe at 12 months.

Regarding the effect of iron fortification in young children on their neurodevelopment, the results made us to refuse our hypothesis, given that we did not see any benefit in the high–Fe group compared with low–Fe group. In fact, the scores obtained in bivariate analysis in both MDI and PDI of Bayley Scales were very similar between the two groups, which prevent us to determine if there was an impact of the supplemental iron dose on neurodevelopment. Previous studies showed contradictory results and some of them, coinciding with the present study, did not observe any positive effect of the supplementation with high doses of iron (within dietary recommendations). In this line, Sungthong et al. [22] found no evidence that iron supplementation could improve school performance in 397 iron–deficient and anemic children in the Southeast Asian. In Turkey, Yalçin et al. [24] reported the lack of benefit on cognitive development in nine–month–old infants after iron supplementation for 3 months. Another study [23] with similar results was conducted in the United Kingdom in 493 healthy children at 18 months of age; the authors did not see any benefit in developmental outcomes in children fed with iron–supplemented formula, but did not exclude the hypothesis about the possibility that some benefit could arise at later ages or in those who were anemic. On the contrary, Lozoff et al. [18] described in a review the benefits observed in mental functioning at 12 months of age after evaluating 1657 healthy Chilean children [30] supplemented with similar iron doses (0.2–1.2 mg/100 mL) to ours. The review also gathered the studies of Friel et al. [15] and Moffat et al. [46] in Canada and Soewondo et al. [47] in Indonesia, who concluded that iron supplementation resulted in beneficial effects for the development of the evaluated children, aged between 9 months and 5 years.

When observing the characteristics of the studies, it seems that the effect of the intervention may be related to the iron status of the children prior to supplementation and the socioeconomic characteristics of the family or environment. In this sense, a recent systematic review [21] concluded that iron supplementation in childhood safely improves the mental and motor performance of young children, especially in low– or middle–income countries. Similarly, the meta–analysis of Sachdev et al. [48] showed a modest improvement on mental development in iron–deficient anemic children above 7 years of age after the iron supplementation. Thus, we suggest that the fact that our study was conducted in well–nourished children with a minute prevalence of ID in a developed country with a high–medium income could underlie the lack of effectiveness of our intervention. In addition, it is worth mentioning that in the present study, serum ferritin was measured at birth and was in the normal range; this indicates the good iron status of the babies at birth which is determined during the prenatal stage. In this sense, given cerebral maturation and the neurological developement of the child take place to a great extent during the prenatal period [49, 50], in healthy children with good iron status at birth, postnatal iron therapy may be unable to change the course of neurodevelopment. Moreover, the age of the evaluation could be another explanation for the disagreement of results in the available literature, as suggested in two Cochrane systematic reviews [26, 27] which recommended performing large randomized controlled trials with long–term follow–up for future investigations.

### Strengths and limitations

The follow–up losses were minimal (6.5%) thanks to the adherence visits at 3 and 9 months and a close monitoring of the infants. Despite that, the short follow–up time was perhaps the main limitation given some studies have been previously found an effect of iron supplementation in child neurodevelopment at later ages. The small sample size was another limitation of the study, which also could reduce the statistical power of our results. In this regard, based on the hypothesis that low doses of iron could be harmful to children’s health, the sample size of high–Fe group was bigger than the low–Fe group. However, a larger low–Fe group would have improved the study and reinforced the obtained results.

Most of the studies published to date were carried out in developing countries or in iron–deficient children, so our results obtained in a developed country, are more appropriate to apply in a non–iron deficient population. Also in contrast to what is common, our intervention compared two suitable doses of iron while most authors have only contrasted the effect of one dose with placebo.

To assess the neurological development of children, we used the BSID–II, which was the current version at the time of the study, although it was later shown to present some errors to evaluate psychomotor development.

## Conclusion

The present study adds to the body of knowledge on the prevalence of ID and IDA in children. It also provides new data on the effect of iron supplementation in children with doses within the dietary recommendations, at the hematological and neurobehavioral level. So, we can conclude that the intervention with infant formula enriched with iron at the maximum dose within the recommended range, from 6 to 12 months of age, did not show any effect on the neurological development of well–nourished children in a developed country at 12 months. Follow–up studies are needed to test for long–term neurodevelopmental improvement.

## Data Availability

The datasets used and/or analysed during the current study are available from the corresponding author on reasonable request.

## Abbreviations

%TS: Transferrin Saturation
12M: 12 months
6M: 6 months
BMI: Body Mass Index
BSID–II: Bayley Scales for Infant Development–Second Edition
Fe: Iron
Hb: Hemoglobin
High–Fe: high–iron group
ID: Iron Deficiency
IDA: Iron Deficiency Anemia
Low–Fe: low–iron group
MCV: Mean Corpuscular Volume
MDI: Mental Development Index
PDI: Psychomotor Development Index
RCT: Randomized Controlled Trial
SES: Socioeconomic Status
SF: Serum Ferritin
STAI: State–Trait Anxiety Inventory

## References

[1] McLean E, Cogswell M, Egli I, Wojdyla D, de Benoist B (2009). Worldwide prevalence of anaemia, WHO vitamin and mineral nutrition information system, 1993-2005. Public Health Nutr.

[2] Hercberg S, Preziosi P, Galan P (2001). Iron deficiency in Europe. Public Health Nutr.

[3] Durá Travé T, Díaz VL (2002). Prevalencia de la deficiencia de hierro en lactantes sanos de 12 meses de edad. An Esp Pediatr.

[4] Arija V, Salas J, Fernández-Ballart J, Marti-Henneberg C (1990). Iron deficiency risk in children: discrepancy between dietary and biochemical assessments. Int J Vitam Nutr Res.

[5] Hallberg L (2001). Perspecives on nutritional iron deficiency. Annu Rev Nutr.

[6] Domellöf M (2011). Iron requirements in infancy. Ann Nutr Metab.

[7] Griffin IJ, Abrams SA (2001). Iron and breastfeeding. Pediatr Clin N Am.

[8] Behrman RE, Kliegman RM, Jenson HB (2004). Enfermedades de la sangre. Anemia fisiológica de la lactáncia. Tratado de Pediatría.

[9] Rao S (1981). Physiological anemia of infancy and anemia of prematurity. Indian J Pediatr.

[10] von Drygalski A, Adamson JW (2013). Iron metabolism in man. JPEN.

[11] Lou J, Mai X, Lozoff B, Felt BT, Kileny PR, Zhao Z (2016). Prenatal iron deficiency and auditory brainstem responses at 3 and 10 months: a pilot study. Hong Kong J Paediatr.

[12] Vaivada Tyler, Gaffey Michelle F., Bhutta Zulfiqar A. (2017). Promoting Early Child Development With Interventions in Health and Nutrition: A Systematic Review. Pediatrics.

[13] Ziegler EE, Nelson SE, Jeter JM (2009). Iron status of breastfed infants is improved equally by medicinal iron and iron-fortified cereal. Am J Clin Nutr.

[14] Gondolf UH, Tetens I, Michaelsen KF, Trolle E (2013). Iron supplementation is positively associated with increased serum ferritin levels in 9-month-old Danish infants. Brit J Nutr.

[15] Friel JK, Aziz K, Andrews WL, Harding SV, Courage ML, Adams RJ (2003). A double-masked, randomized control trial of iron supplementation in early infancy in healthy term breast-fed infants. J Pediatr.

[16] Eichler K, Wieser S, Rüthemann I, Brügger U (2012). Effects of micronutrient fortified milk and cereal food for infants and children: a systematic review. BMC Public Health.

[17] Mcdonagh MS, Blazina I, Dana T, Cantor A, Bougatsos C (2015). Screening and routine supplementation for Iron deficiency Anemia: a systematic review. Pediatrics.

[18] Lozoff B, Beard J, Connor J, Felt B, Georgieff M, Schallert T (2006). Long-lasting neural and behavioral effects of iron deficiency in infancy. Nutr Rev.

[19] Angulo-Barroso RM, Li M, Santos DC, Bian Y, Sturza J, Jiang Y (2016). Iron Supplementation in Pregnancy or Infancy and Motor Development: A Randomized Controlled Trial. Pediatrics.

[20] Lind T, Lönnerdal B, Stenlund H, Gamayanti IL, Ismail D, Seswandhana R (2004). A community-based randomized controlled trial of iron and zinc supplementation in Indonesian infants: effects on growth and development. Am J Clin Nutr.

[21] Low M, Farrell A, Biggs BA, Pasricha SR (2013). Effects of daily iron supplementation in primary-school-aged children: systematic review and meta-analysis of randomized controlled trials. CMAJ.

[22] Sungthong R, Mo-suwan L, Chongsuvivatwong V, Geater AF (2004). Once-weekly and 5-days a week iron supplementation differentially affect cognitive function but not school performance in Thai children. J Nutr.

[23] Morley R, Abbott R, Fairweather-Tait S, MacFadyen U, Stephenson T, Lucas A (1999). Iron fortified follow on formula from 9 to 18 months improves iron status but not development or growth: a randomised trial. Arch Dis Child.

[24] Yalçin S, Yurdakök K, Açikgöz D, Özmert E (2000). Short-term developmental outcome of iron prophylaxis in infants. Pediatr Int.

[25] Lozoff B, Castillo M, Clark KM, Smith JB (2012). Iron-fortified vs low-iron infant formula: developmental outcome at 10 years. Arch Pediatr Adolesc Med.

[26] Martins S, Logan S, Gilbert R (2001). Iron therapy for improving psychomotor development and cognitive function in children under the age of three with iron deficiency anaemia. Cochrane Db Syst Rev.

[27] Wang B, Zhan S, Gong T, Lee L (2013). Iron therapy for improving psychomotor development and cognitive function in children under the age of three with iron deficiency anaemia. Cochrane Db Syst Rev.

[28] Golz A, Netzer A, Goldenberg D, Westerman ST, Westerman LM, Joachims HZ (2001). The association between iron-deficiency anemia and recurrent acute otitis media. Am J Otol.

[29] Domellöf M, Braegger C, Campoy C, Colomb V, Decsi T, Fewtrell M (2014). Iron requirements of infants and toddlers. J Pediatr Gastroenterol Nutr.

[30] Lozoff B, De Andraca I, Castillo M, Smith JB, Walter T, Pino P (2003). Behavioral and developmental effects of preventing Iron-deficiency Anemia in healthy full-term infants. Pediatrics.

[31] Hollingshead AB (2011). Four factor index of social status. Yale J Sociol.

[32] Spielberger CD, Gorsuch RL, Lushene RE, Buela-Casal G, Cubero NS, Guillén-Riquelme A (2011). STAI: Cuestionario de Ansiedad Estado-Rasgo.

[33] Fairbanks VF (1999). Biochemical aspects of haematology. Tietz textbook of clinical chemistry.

[34] Abidin RR (1995). The parenting stress index.

[35] Center for disease control and Prevention (1998). Recommendations and Reports. Recommendations to prevent and control iron deficiency in the United States. MMWR.

[36] World Health Organization. Iron Deficiency Anaemia: Assessment, Prevention and Control, A guide for program managers. Control. 2001:114 http://apps.who.int/iris/bitstream/10665/66914/1/WHO_NHD_01.3.pdf?ua=1.

[37] Baker RD, Greer FR, The Committee On Nutrition (2010). Diagnosis and prevention of Iron deficiency and Iron-deficiency Anemia in infants and young children (0−3 years of age). Pediatrics.

[38] Bayley N (1993). Manual for the Bayley Scales of Infant Development.

[39] Dallman PR (1995). Exámenes de laboratorios para el diagnóstico de la deficiencia de hierro en el lactante y en la primera infancia. An Nestle.

[40] Saarinen UM, Siimes MA (1978). Developmental changes in red blood cell counts and indices of infants after exclusion of iron deficiency by laboratory criteria and continuous iron supplementation. J Pediatr.

[41] Siimes MA, Addiego JE, Dallman PR (1974). Ferritin in serum: diagnosis of Iron deficiency and Iron overload in infants and children. Blood.

[42] Michaelsen KF, Milman N, Samuelson G (1995). A longitudinal study of iron status in healthy Danish infants: effects of early iron status, growth velocity and dietary factors. Acta Paediatr.

[43] Domellöf M, Lind T, Lönnerdal B, Persson LÅ, Dewey KG, Hernell O (2008). Effects of mode of oral iron administration on serum ferritin and haemoglobin in infants. Acta Paediatr.

[44] Pasricha SR, Hayes E, Kalumba K, Biggs BA (2013). Effect of daily iron supplementation on health in children aged 4-23 months: a systematic review and meta-analysis of randomised controlled trials. Lancet Glob Health.

[45] Gera T, Sachdev HPS (2002). Effect of iron supplementation on incidence of infectious illness in children: systematic review. BMJ.

[46] Moffatt ME, Longstaffe S, Besant J, Dureski C (1994). Prevention of iron deficiency and psychomotor decline in high-risk infants through use of iron-fortified infant formula: a randomized clinical trial. J Pediatr.

[47] Soewondo S, Husaini M, Pollitt E (1989). Effects of iron deficiency on attention and learning processes in preschool children: Bandung. Indonesia Am J Clin Nutr.

[48] Sachdev H, Gera T, Nestel P (2005). Effect of iron supplementation on mental and motor development in children: systematic review of randomised controlled trials. Public Health Nutr.

[49] Hernández-Martínez C, Canals J, Aranda N, Ribot B, Escribano J, Arija V (2011). Effects of iron deficiency on neonatal behavior at different stages of pregnancy. Early Hum Dev.

[50] Kocevska D, Verhoeff ME, Meinderts S, Jaddoe VWV, Verhulst FC, Roza SJ (2018). Prenatal and early postnatal measures of brain development and childhood sleep patterns. Pediatr Res.

